# Relationships between apparent cortical thickness and working memory across the lifespan - Effects of genetics and socioeconomic status

**DOI:** 10.1016/j.dcn.2021.100997

**Published:** 2021-08-08

**Authors:** Stine K. Krogsrud, Athanasia M. Mowinckel, Donatas Sederevicius, Didac Vidal-Piñeiro, Inge K. Amlien, Yunpeng Wang, Øystein Sørensen, Kristine B. Walhovd, Anders M. Fjell

**Affiliations:** a Center for Lifespan Changes in Brain and Cognition, Department of Psychology, University of Oslo, 0317, Oslo, Norway; b Department of Radiology and Nuclear Medicine, Oslo University Hospital, 0372, Oslo, Norway

**Keywords:** ABCD, the Adolescent Brain Cognitive Development, CT, cortical thickness, GAMM, Generalized additive mixed models, GCA, general cognitive ability, LCBC, the Center for Lifespan Changes in Brain and Cognition, LME, linear mixed effect, MRI, magnetic resonance imaging, NIH, the National Institutes of Health, SES, socioeconomic status, SNP, single nucleotide polymorphism, WASI, Wechsler Abbreviated Scale of Intelligence, WM, working memory, WPPSI, Wechsler Preschool and Primary Scale of Intelligence, Cortical thickness, Digit span, Development, Heritability, Lifespan, Working memory

## Abstract

Working memory (WM) supports several higher-level cognitive abilities, yet we know less about factors associated with development and decline in WM compared to other cognitive processes. Here, we investigated lifespan changes in WM capacity and their structural brain correlates, using a longitudinal sample including 2358 magnetic resonance imaging (MRI) scans and WM scores from 1656 participants (4.4–86.4 years, mean follow-up interval 4.3 years). 8764 participants (9.0–10.9 years) with MRI, WM scores and genetic information from the Adolescent Brain Cognitive Development study were used for follow-up analyses. Results showed that both the information manipulation component and the storage component of WM improved during childhood and adolescence, but the age-decline could be fully explained by reductions in passive storage capacity alone. Greater WM function in development was related to apparent thinner cortex in both samples, also when general cognitive function was accounted for. The same WM-apparent thickness relationship was found for young adults. The WM-thickness relationships could not be explained by SNP-based co-heritability or by socioeconomic status. A larger sample with genetic information may be necessary to disentangle the true gene-environment effects. In conclusion, WM capacity changes greatly through life and has anatomically extended rather than function-specific structural cortical correlates.

## Highlights

• Sub-components of working memory (WM) showed different lifespan trajectories.
• WM capacity was related to apparent thinner cortex during childhood.
• The WM-thickness effect could not be accounted for by general cognitive abilities.
• The WM-thickness relationship was not mediated by genetics or socioeconomic status.

## 1 Introduction

Working memory (WM), the set of mental processes holding limited information in a temporarily accessible state, may play a part in the emergence of several higher-level cognitive functions (Adams and Hitch, 1997; Baddeley, 1986; Nelson et al., 2000). WM is involved in passive storage of information (short-term memory) and in manipulating and using that information while holding it in mind (Gathercole et al., 2004). It has been argued that WM acts as a bridge between perception, long-term memory and action (Froudist-Walsh et al., 2018). WM capacity predicts individual differences in other intellectual abilities and performance changes across the lifespan (Cowan et al. 2005; Gathercole et al., 2004; Klingberg, 2006; Østby et al., 2011). Changes in WM capacity may therefore be a contributing factor to developmental and aging-related changes in other cognitive functions. A recent meta-analysis showed that change in WM capacity loaded 0.73 on g-factor change in middle-aged and older adults (Tucker-Drob et al., 2018), demonstrating that WM is one of several fundamental cognitive processes that may contribute to decline in global cognitive function in aging.

WM capacity increases during childhood and adolescence (Gathercole et al., 2004; Klingberg, 2006), and decreases in later adulthood (Nyberg et al., 2012, Park et al., 2002). However, compared to for instance episodic memory, we know less about onset of decline in WM performance. Accurately describing the trajectory for change in WM capacity throughout life calls for a combined longitudinal and cross-sectional lifespan approach. Describing the continuous changes in verbal WM capacity through development, adulthood, and aging was the first goal of the present study. Here, we included trajectories for subcomponents of WM namely the information manipulation component and the more passive storage component.

The second goal was to explore structural brain correlates for WM changes by investigating the relationship between WM and cortical thickness change through the lifespan. Apparently thinner cortex has been found to be related to better cognitive performance in children and adolescents in a variety of domains (Schnack et al., 2015; Squeglia et al., 2013; Tamnes et al., 2011; Østby et al., 2012), including WM (Darki and Klingberg, 2015; Tamnes et al., 2010; Østby et al., 2011). In older adults, reductions in thickness have been related to cognitive decline (for a review, see (Fjell and Walhovd, 2010)). Here, we tested whether verbal WM function was related to apparent cortical thickness in a longitudinal lifespan sample. We hypothesized that less apparent cortical thickness would be related to better function during childhood and adolescence, and the opposite relationship in adulthood and aging. A large cross-sectional developmental sample from the Adolescent Brain Cognitive Development (ABCD) study was included for replication purposes, using a different WM test, hence also serving as a validation of the test-specificity of the effects.

The third goal was to estimate single nucleotide polymorphism (SNP) co-heritability for the WM-thickness relationship in ABCD. Twin studies have shown non-zero heritability for cortical thickness (Joshi et al., 2011; Kremen et al., 2010; Rimol et al., 2010), and regional thickness seems to be affected by unique genetic influences not shared with overall cortical thickness (Eyler et al., 2012). Within a smaller twin sample, moderate heritability for spatial WM has been found (Zhou et al., 2018). Intelligence and changes in cortical thickness has been found to be co-heritable (Brans et al., 2010), but no study has to our knowledge investigated the co-heritability for WM-thickness relationships. To complement the genetic analyses, we tested whether socioeconomic status (SES) influenced the WM-thickness relationship. SES inequalities have been associated with differences in executive function, such as WM (Lawson, 2018). Recently, Judd et al. (2020) found that a polygenic score for educational attainment and parental education were independently related to development of cortical surface area and WM, but not to cortical thickness. We hypothesized independent genetic and SES effects on the WM-thickness relationship, but this hypothesis is speculative, as there are few previous studies addressing this.

## 2 Material and methods

### 2.1 LCBC lifespan sample

Participants were drawn from the database of the Center for Lifespan Changes in Brain and Cognition (LCBC), consisting of four longitudinal sub-projects; The Norwegian Mother and Child Cohort - Neurocognitive Study (MoBa) (Magnus et al., 2006; Walhovd et al., 2016), Neurocognitive Development (Tamnes et al., 2013a, b), Neurocognitive Plasticity (de Lange et al., 2018), and Biological Predictors of Memory (Storsve et al., 2014). All relevant aspects of the data collection and handling, including participant informed consent, were approved by the regional ethical board South (https://helseforskning.etikkom.no). There were a total of 2041 available participants, with the age range of 4.1−93.3. The sample was reduced to those with valid Digit Span data (forwards and backwards) and who had completed at least one magnetic resonance imaging (MRI) examination. Adults were further excluded if scoring less than 26 on the Mini-Mental State Examination (Folstein et al., 1975), and/or if scoring more than 20 on either the Beck Depression Inventory (Beck, 1987), and/or the Geriatric Depression Scale. Adult participants were screened using a standardized health interview prior to inclusion in the study. All participants were screened for conditions assumed to affect CNS function (e.g. neurological disorders, epilepsy, stroke, psychiatric disorders or treatment). Participants with a history of self or parent-reported neurological or psychiatric conditions, including clinically significant stroke, serious head injury, untreated hypertension and diabetes within the last two years, were excluded. Further, participants reporting worries concerning their cognitive status, including memory function, were excluded. MRI scans were evaluated by a neuroradiologist and required to be free of significant injuries or conditions. After applying these criteria, the number of unique participants was reduced to 1656, of whom 1011 were female, with in total 2358 MRI scans and Digit Span tests completed. 1483 participants had 1 visit, 649 had 2 visits, 223 had 3 visits, 2 had 4 visits, and 1 had 5 visits. Mean interval between visits was 4.3 years (range: 1.25–10.98). Full scale IQ was calculated from the maximum number of subtests available (either 2 or 4 subtests) of Wechsler Abbreviated Scale of Intelligence (WASI) (Wechsler, 1991) for participants above 6.5 years of age. For participants below or equal 6.5 years of age, full scale IQ was calculated as the mean of Verbal and Performance IQ from four subtests (Similarities, Vocabulary, Block Design and Matrix Reasoning) of Wechsler Preschool and Primary Scale of Intelligence (WPPSI) (Wechsler, 2002). In addition, general cognitive ability (GCA) scores was calculated for all participants across the entire lifespan. The GCA was based on raw scores for matrix reasoning and vocabulary from WASI and WPPSI. The raw scores from WPPSI were then transformed into WASI equivalent scores by estimating age-slopes for both test versions, and then these parameters were used to re-scale the WPPSI scores according to procedures previously described (Fjell et al., 2019). Participant characteristics for the ﬁnal sample are provided in Table 1.

**Table 1:** LCBC sample descriptives.

							Longitudinal data	
Measure	Mean	SD	Min	Max	N	(F/M)	N	Interval
**LCBC Lifespan**								
Age	27.33	20.76	4.42	86.36	2358	(1403/955)	1275	4.48
IQ	113.02	11.53	73.00	146.00	2356	(1401/955)	1274	4.48
GCA	38.87	10.87	4.62	56.00	2358	(1403/955)	1275	4.48
DS For	8.98	2.24	3.00	16.00	2358	(1403/955)	1275	4.48
DS Back	6.27	2.21	0.00	14.00	2358	(1403/955)	1275	4.48

**LCBC Development**								
Age	12.00	4.61	4.42	26.66	1195	(614/581)	881	4.50
IQ	109.34	109.34	11.75	143.00	1194	(613/581)	880	4.50
GCA	31.65	31.65	10.53	56.00	1195	(614/581)	881	4.50
DS For	8.28	8.28	2.12	15.00	1195	(614/581)	881	4.50
DS Back	5.49	5.49	2.05	13.00	1195	(614/581)	881	4.50

**LCBC Adults**								
Age	33.98	11.75	20.04	64.60	876	(612/264)	270	4.14
IQ	115.39	9.47	83.00	140.00	875	(611/264)	270	4.14
GCA	46.52	3.86	33.50	56.00	876	(612/264)	270	4.14
DS Back	9.93	2.11	5.00	16.00	876	(612/264)	270	4.14
DS Back	7.29	2.05	2.00	14.00	876	(612/264)	270	4.14

**LCBC Older adults**								
Age	70.85	4.85	60.02	86.36	287	(177/110)	124	5.07
IQ	121.06	10.27	79.00	146.00	287	(177/110)	124	5.07
GCA	45.60	4.72	27.00	55.00	287	(177/110)	124	5.07
DS For	9.03	2.03	5.00	15.00	287	(177/110)	124	5.07
DS Back	6.41	2.05	2.00	13.00	287	(177/110)	124	5.07

Summary for the total LCBC Lifespan sample. DS For = Digit Span Forwards, DS Back = Digit Span Backwards, GCA = general cognitive ability. N equals number of observations and not number of participants. Interval is the mean of the interval for the longest follow-up per person. Participants in the MoBa sample below 6.5 years of age were tested with the WPPSI matrix and similarities version, while the rest of the LCBC Lifespan sample used the WASI. IQ is calculated from the maximum number of subtests available for WASI (either 2 or 4 subtests), while WPPSI is calculated as the mean of Verbal and Performance IQ. GCA score is based on raw scores for matrix reasoning and vocabulary from WASI and WPPSI. The raw scores from WPPSI were then transformed into WASI equivalent scores by estimating age-slopes for both test versions, and then these parameters were used to re-scale the WPPSI scores.

### 2.2 ABCD sample

The Adolescent Brain Cognitive Development (ABCD) study is an ongoing project funded by the National Institutes of Health (NIH) (https://abcdstudy.org). This is a multisite, longitudinal study designed to recruit more than 10000 children aged 9–10 and follow them over 10 years into early adulthood. The participants (including 1600 twins) were recruited at 21 different US sites. For the current report, the ABCD dataset release 2.0.1 was used (Yang and Jernigan, 2019), and DOIs can be found at https://nda.nih.gov/study.html?id=817. There were 9053 participants available after excluding 2105 twins and 30 triplets. Of the 8896 subjects with List Sorting WM Test scores, 8764 participants (4160 females) had MRI data with quality check *OK* and were included in the analyses. Full scale IQ (ABCD data-nihtbx_totalcomp_uncorrected) was entered as a Total Cognition Composite score (a combination of both Crystallized and Fluid Intelligence scores) (Heaton et al., 2014; Michelini et al., 2019) from the NIH Toolbox. Participant characteristics for the ﬁnal sample are provided in Table 2.

**Table 2:** ABCD sample descriptives.

Measure	Mean	SD	Min	Max	N (F/M)
Age	9.88	0.63	9.00	10.92	8896 (4226/4667)
IQ Fullscale	86.28	9.19	44.00	117.00	8857 (4203/4651)
List recall	96.78	12.09	36.00	136.00	8896 (4226/4667)

Summary for the ABCD sample. IQ measures are uncorrected.

Among the 8764 participants, 7929 also had genotypes from doi:10.15154/1503209. Briefly, samples were genotyped by the Affymetrix NIDA SmokeScreen Array, which contains 733293 SNPs. Raw genotypes were quality controlled by the ABCD consortium according to the recommendation from the Ricopili pipeline (Lam et al., 2019). SNPs having a call-rate bellow 0.99 across all the ABCD sample were removed, and participants having missing-rate larger than 20 % and/or having potential contamination were excluded. Before estimating the co-heritability using genotype data, related ABCD participants (closer than the third degree, i.e., kinship coefficient >0.0625) were removed using the PLINK program (Chang et al., 2015). For each pair of related participants, one was randomly included in the analysis. In total, 7247 children remained. Socioeconomic status was estimated based on parental education and parental income. Education (ABCD data-field high.educ) was entered as the highest education of parents (1: < HS Diploma, 2: HS Diploma/GED, 3: some college, 4: Bachelor, 5: Post Graduate Degree). Income (ABCD data-field demo_comb_income_p) was entered as the parents combined income, recoded to the middle of each income category.

### 2.3 Working memory assessment

#### 2.3.1 LCBC lifespan sample

Verbal WM was assessed with diﬀ ;erent Digit Span versions, all with the same amount of sequences and sequence lengths. The versions used were the Wechsler Intelligence Scale for Children Third Edition (WISC-III) (Wechsler, 1991), Wechsler Adult Intelligence Scale - Third and Fourth Edition (WAIS-III/WAIS-IV) (Wechsler, 1997), Wechsler Memory Scale–Revised (WMS–R) (Wechsler, 1987), and two versions developed in-house with a random number generator. The in-house versions were made to avoid retest effects. Participants were verbally presented with numeric sequences of increasing length. In the ﬁrst part of the test, they were required to repeat the digits in the same order as presented (Digit Span Forwards). In the second part, they were asked to repeat the digits in reversed presentation order (Digit Span Backwards). The length of the digit sequences increased every other sequence. The stop criterion was two wrong answers within a pair of equal length. One point was awarded for each correctly repeated sequence of digits. Digit Span Forwards is assumed to be dependent on simple storage span, as held in the phonological loop, while Digit Span Backwards is thought to be more reﬂective of executive control of WM (Engle, Tuholski, Laughlin, & Conway, 1999; Gathercole et al., 2004). Both Digit Span Forwards and Digit Span Backwards scores were used in the present study, and Digit Span Total (forwards + backwards) and ratio scores (backwards/forwards) were calculated. Ratio scores are believed to more purely reflect the active information manipulation component of WM because the short-term storage component is controlled for.

#### 2.3.2 ABCD sample

The List Sorting WM Test is derived from the NIH Toolbox, and is a sequencing task requiring children to sort information and sequence it. Items are presented both visually and auditorily. The participants are presented with a series of illustrated pictures, each depicting an item (e.g., an animal) on a tablet, along with their auditory names. Participants are instructed to remember the stimuli and to repeat them verbally to the examiner in the order of size, from smallest to largest. The number of objects in a series increases on successive items thereby taxing the WM system when longer sequences need to be remembered. Furthermore, the task starts with a “1-list” version where the participants have to sequence one type of stimuli (e.g., “animals” or “food”) according to size order and then increases to a “2-list” version where two types of stimuli have to be sequenced, each in size order. The obtained score is the sum of the total number of items correctly recalled and sequenced on 1-list and 2-list. In the 2-list version, the WM load is increased substantially as the stimuli are presented from two categories (animals and food) and the participants have to track and organize stimuli from both categories and report by size the items from one category (i.e., animals) and then the other category by size (i.e., food). It is this “dual” tracking and processing information that increases the WM load of the task (Weintraub et al., 2013).

### 2.4 MRI acquisition and preprocessing

#### 2.4.1 LCBC lifespan sample

Imaging data were acquired from three different Siemens scanner models in Norway: Avanto 1.5 T (T) at St. Olavs Hospital in Trondheim, and Avanto 1.5 T, Skyra 3 T and Prisma 3 T at Oslo University Hospital Rikshospitalet. A total of 992 scans were collected on Avanto scanners, of which 226 were collected in Trondheim. On the two Avanto scanners, identical T1 weighted MPRAGE’s were collected with the following parameters: TR: 2400 ms, TE: 3.61 ms, TI: 1000 ms, flip angle: 8 °, slice thickness: 1.2 mm, FoV: 240 × 240, and 160 slices. There were 819 scans collected at the Skyra with the following parameters: TR: 2300 ms, TE: 2.98 ms, TI: 850 ms, flip angle: 8 °, slice thickness: 1 mm, FoV: 256 × 256, and 176 slices. On the Prisma, there were 321 scans with the following parameters: TR: 2400 ms, TE: 2.22 ms, TI: 1000 ms, flip angle: 8 °, slice thickness: 0.8 mm, FoV: 240 × 256, and 208 slices. For the youngest children, integrated parallel acquisition techniques (iPAT) were used, acquiring multiple T1 scans within a short scan time, enabling us to discard scans with residual movement. Previous studies have shown that accelerated imaging does not introduce measurement bias in surface-based measures when using FreeSurfer for image analysis, compared with a standard MPRAGE protocol with otherwise identical voxel dimensions and sequence parameters (Wonderlick et al., 2009). All scans were visually rated for movement, and only scans with rating 1–2 on a 4-point scale were included (no visible or only very minor possible signs of movement), as movement is a major concern (Reuter et al., 2015). In all cases, the single best scan was used for processing.

MRI data were processed and analyzed with FreeSurfer 6.0 (Dale et al., 1999; Fischl et al., 2004, 1999a; Fischl et al., 1999b) (http://surfer.nmr.mgh.harvard.edu/), using the longitudinal stream on all participants (Reuter et al., 2012). This procedure yields a measure of cortical thickness for each person at each point on the reconstructed surface and is capable of detecting sub-millimeter differences between groups (Fischl and Dale, 2000; Kuperberg et al., 2003; Rosas et al., 2002). The processing steps include removal of non-brain tissue (Segonne et al., 2004), automated Talairach transformation, and intensity correction (Sled et al., 1998). Intensity and continuity information from the 3D volume are used in segmentation and deformation procedures to reconstruct a gray/white and gray/cerebrospinal fluid boundary throughout the brain (Dale et al., 1999; (Fischl, 2002); (Fischl, 2004)). Cortical surfaces then undergo inflation, registration to a spherical atlas, and identification of gyral and sulcal regions (Fischl et al., 2004a; (Desikan, 2006)). Specific advantages of the longitudinal stream include that an unbiased within-subject template space and image (Reuter and Fischl 2011) is created using robust, inverse consistent registration (Reuter et al., 2010). Several of the usual processing steps, such as skull stripping, Talairach transforms, atlas registration as well as spherical surface maps and parcellations are then initialized with common information from the within-subject template, significantly increasing reliability and statistical power (Reuter et al., 2012). Smoothing using a circularly symmetric Gaussian kernel with a full width at half maximum (FWHM) of 15 mm was used for cortical thickness maps.

Different scanners have been found to yield different thickness estimates but not to bias the correlation with external measures such as cognitive test scores or skew the rank-order between the participants (Dickerson et al., 2008). Thus, we expected that including scanner as a covariate of no interest in the analyses would remove most of the effects on cortical thickness estimates resulting from the use of different scanners. To test this assumption, 307 participants were scanned both on the 1.5 T Avanto and on the 3 T Skyra on the same day in Oslo, and the results were compared. First, mean thickness was compared between scanners by paired-samples *t*-test. There were no significant differences in estimated mean cortical thickness between scanners (t = -1.70, p = 0.091). In addition, mean cortical thickness was plotted as a function of scanner. The plot showed good rank order correspondence between the scanners (supplementary material, Fig. 1), and the paired samples correlation was 0.91. Further, sample density was plotted as a function of scanner, which shows overlap between the age-range of the samples from the different scanner models (supplementary material, Fig. 1).

#### 2.4.2 ABCD sample

Brain imaging data were collected and processed by the ABCD study using FreeSurfer 5.3. Brain imaging data were collected across 21 sites on 3 T scanners (Siemens Prisma, Siemens Corp., Erlanger, Germany), GE Discovery MR750 (GE Healthcare, Chicago, IL), and Philips Achieva (Philips, Amsterdam, the Netherlands). Acquisition parameters are listed in Casey et al. (2018) and at https://abcdstudy.org/images/Protocol_Imaging_Sequences.pdf. Imaging preprocessing steps are described in Hagler et al. (2019), and included gradient nonlinearity distortion correction, intensity inhomogeneity correction, and registration and resampling to a custom atlas brain with 1.0 mm isotropic voxels. Brain volumes were computed from the preprocessed T1 images using FreeSurfer version 5.3.0.

### 2.5 Statistical analysis

Generalized additive mixed models (GAMM) were run with R version 4.0.0 (R Core Team, 2020) with the package *mgcv*, version 1.8–3 (Wood, 2017) to estimate age-trajectories for Digit Span scores for the LCBC Lifespan sample. The smoothness of the age and time-curve was estimated as part of the model ﬁt, and the resulting eﬀ ;ective degrees of freedom (edf) was taken as a measure of deviation from linearity. GAMMs are well-suited to map trajectories of cognitive and brain variables which can be assumed to be non-linear (Fjell et al., 2010; Sørensen et al., 2020). For the Digit Span test, models were run for all four Digit Span scores (backwards, forwards, total and ratio). Participant intercept was included as a random eﬀ ;ect variable, and a smooth term was used for age. Sex and number of test repetitions to control for practice effects were included as covariates.

For stratified analyses, the LCBC Lifespan sample was divided into three age groups (development, adults and older adults) based on qualitative inspection of the GAMM fits estimating age-trajectories for Digit Span Backwards scores. The developmental group consisted of 702 participants with 1195 observations, the adult group consisted of 740 participants with 876 observations, and the older adults consisted of a group with 214 participants and 287 observations. Correlations between all four Digit Span scores were run across the lifespan and for the three age groups separately. For the developmental group, correlations were also run between Digit Span Backwards and GCA scores. Also, three equally sized age-corrected performance groups were created by fitting a linear model predicting performance on the Digit Span task using age and Digit Span repetition as predictors.

GAMMs were also run to estimate an age-trajectory for mean cortical thickness for the LCBC Lifespan sample, covaried for sex and scanner.

To test the relationship between WM performance and cortical thickness in the LCBC Lifespan sample, vertex-wise linear mixed effect (LME) models were run with FreeSurfer 6.0 (Bernal-Rusiel et al., 2013a, b) for the three age-groups separately. We used random intercepts to account for correlation between repeated measures of thickness in the same individual. This takes advantage of the longitudinal observations, while still utilizing the increased statistical power of each single measurement. The LMEs were used to test the eﬀ ;ects of Digit Span Backwards scores on cortical thickness, with sex, age and scanner as linear covariates. As Digit Span Backwards scores presumably best reflects both the storage and manipulation components of WM, this was our main focus. However, additional LME models were run to test the effects of (1) Digit Span Total scores (forwards + backwards), (2) Digit Span Forwards scores and (3) ratio scores (backwards/forwards) on cortical thickness for the three age-groups separately.

For the LCBC developmental group, two supplementary analyses were run to test the eﬀ ;ects of Digit Span Backwards scores on cortical thickness with GCA scores and mean thickness as additional covariates to control for a general cognitive function effect and a global anatomical effect, respectively. The backwards score was chosen as this presumably best reflects both the storage and the manipulation aspects of WM. Supplementary analyses were also run to test the relationship between (1) Digit Span Backwards scores and volume, and (2) Digit Span Backwards scores and surface area for all three age groups separately. The same LMEs were then re-run with mean volume and mean surface area as additional covariates. To control for multiple comparisons across space, all surface results for both the LCBC and ABCD samples were thresholded by Monte Carlo simulations using a cluster-forming threshold of 0.01 and a corrected cluster p-value of 0.05 (Hagler et al., 2006).

For the ABCD sample, a general linear model (GLM) was run with FreeSurfer 6.0 (Bernal-Rusiel et al., 2013b) to test the cross-sectional relationship between cortical thickness and WM performance, with sex, age and ethnicity as covariates (see Table 1 in Supplementary material for an overview of self-reported ethnicity). In addition, correlation between estimated WM-thickness effects at each vertex in the LCBC and the ABCD samples was computed to assess how similar the distribution of effects was between the samples. For supplementary analyses, the GLMs were also run to test the relationship between (1) WM and volume, and (2) WM and surface area with sex, age and ethnicity as covariates.

#### 2.5.1 Co-heritability and socioeconomic status

To estimate how much of the phenotypic relationship between WM and cortical thickness was due to common genetic influence, co-heritability was calculated for each anatomical region where cortical thickness was associated with WM development in the ABCD sample. The genetic ancestry factors were computed using the principal component analysis framework with thinned SNPs using PLINK. The following parameters were used: --maf 0.05, --geno 0.05, --hwe 1e-5, and –indep-pairwise 100 50 0.1. The software GCTA (Yang et al., 2011) was used to estimate the co-heritability between cortical thickness and WM. Age at scan, sex and the top 10 genetic ancestry factors were included as covariates during the estimation.

Further, we tested whether the inclusion of two SES variables (parental education and parental income) affected the observed WM-thickness relationships in ABCD. Two linear regression models were run, both controlling for the effect of age, sex and ethnicity, and one with parental education and parental income as additional covariates. The model fit of regression models with and without SES as predictors were then compared.

## 3 Results

### 3.1 Working memory performance across the lifespan in the LCBC sample

**Fig. 1:**
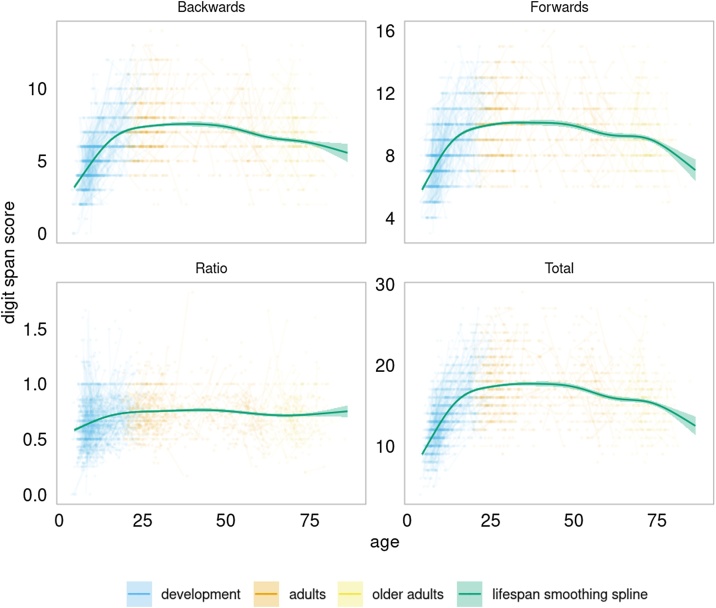
Working memory performance across the lifespan. LCBC Lifespan sample (4.4–86.4 years). Lines between dots connect longitudinal measurements. For all Digit Span measurements, the lifespan trajectory was estimated by a smoothing curve over age with a GAMM including sex and test repetitions as covariates. Backwards = Digit Span Backwards scores, Forwards = Digit Span Forwards scores, Ratio = ratio scores (backwards/forwards) and Total = Digit Span Total scores (forwards + backwards).

The age-trajectories for WM performance for the LCBC Lifespan sample are presented in Fig. 1. All WM measures were significantly related to age, forming non-linear trajectories (backwards: edf = 7.18, F = 94.97, p < .001/ forwards: edf = 7.62, F = 77.51, p < .001/ total: edf = 7.75, F = 117.85, p < .001/ ratio: edf = 7.51, F = 17.43, p < .001). Backwards, forwards and total score showed very similar trajectories with increases until early 20′s, followed by a period of relative stability in adulthood, and then accelerated decline from about sixty years of age. In contrast, the ratio (backwards/forwards) trajectory was characterized by an early developmental eﬀ ;ect, with no notable age-change after about 20 years. The three age groups (development, adults and older adults) were defined based on qualitative inspection of the age-trajectories for Digit Span Backwards scores, and the baseline age cut-oﬀ ; for the adult group was > 16.9 years of age, representing the initiation of the reduction in improvement, which may be a more interesting developmental marker than peaks or dips (see also (Fjell et al., 2013)). For the older adults, the baseline age cut-oﬀ ; was > 59.9 years of age, representing an accelerated decrease in WM performance.

**Fig. 2:**
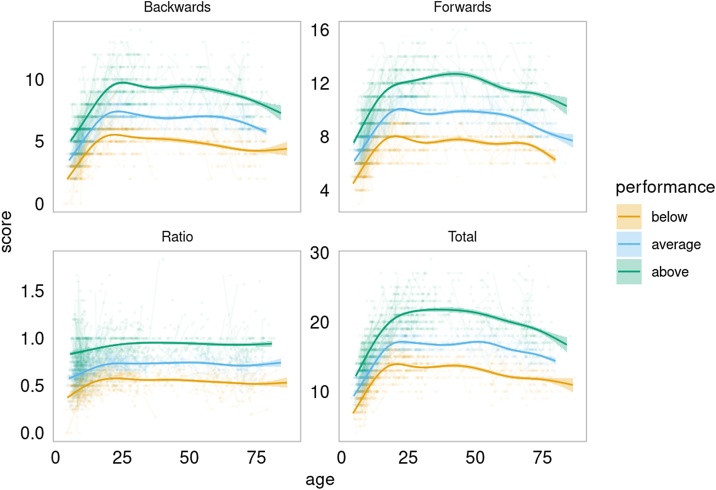
Working memory performance across lifespan according to performance group. LCBC Lifespan participants were divided into three performance groups. The “below” group scored in the bottom 33.3 percentile, the “above” group are participants in the top 66.6 percentile, and “average” the remaining participants.

The correlation structure for the Digit Span measures is shown in Table 3. These were very similar across the three age-groups. Backwards and forwards correlated 0.61, suggesting that they to some degree are measuring a similar underlying construct. In the developmental group, Digit Span Backwards and GCA correlated 0.61, highlighting that WM performance is associated with several cognitive processes. For visualization purposes, participants were divided into performance groups based on their Digit Span scores for backwards, forwards, ratio and total. The smoothing curves showed rather parallel age trajectories, but as the performance groups are divided by function at every age, caution should be taken when interpreting them (see Fig. 2). To formally test the relationship between Digit Span performance (level) and change in performance (slope), we correlated the slope with the centercept (mean across timepoints) for Digit Span Total in the full sample of participants with at least two timepoints, residualized on age by use of GAM. This yielded a correlation of 0.035 (p = .40), confirming the impression from Fig. 2 that Digit Span change across time is not related to the initial starting level of performance.

**Table 3:** Correlation matrix of the Digit Span measures.

		Backwards	Ratio	Total
Lifespan	Forwards	0.61	−0.41	0.9
	Backwards		0.67	0.9
	Ratio			0.29
Development Adults	Forwards	0.57	−0.17	0.89
	Backwards		0.68	0.88
	Ratio			0.28
Adults	Forwards	0.54	−0.27	0.88
	Backwards		0.64	0.87
	Ratio			0.21
Older adults	Forwards	0.53	−0.2	0.87
	Backwards		0.69	0.88
	Ratio			0.29

Digit Span *Total* is the sum of backwards and forwards, while *Ratio* is the ratio backwards over forwards. The table display the correlation matrices for each age group, as well across the entire sample.

### 3.2 Relationship between working memory and cortical thickness: vertex-wise linear mixed models

**Fig. 3:**
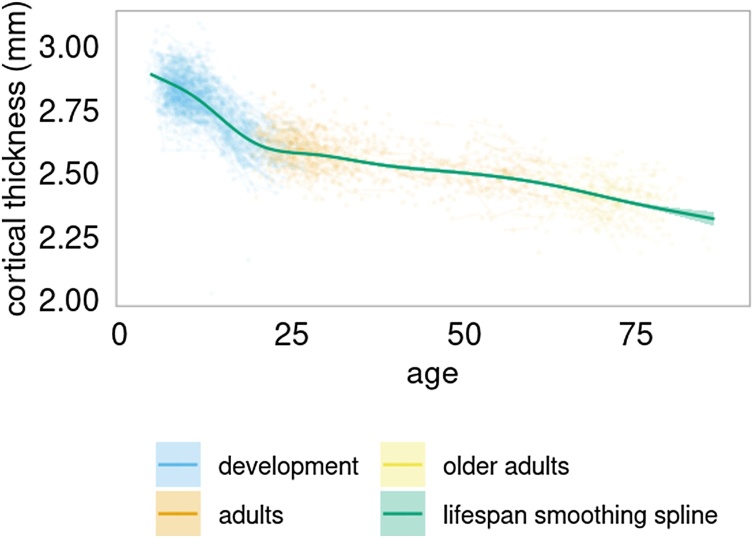
Global cortical thickness across the lifespan. LCBC Lifespan sample (4.4–86.4 years). The GAMM controlled for the effect of sex and scanner as covariates. The baseline age cut-oﬀ ; for the adult group was > 16.9 years of age, and > 59.9 years of age for the old adults.

Age-trajectories across the lifespan for mean apparent cortical thickness showed a non-linear decrease from 4.4–86.4 years, with a more rapid decrease for the developmental period compared to adulthood (see Fig. 3).

#### 3.2.1 LCBC lifespan sample

**Fig. 4:**
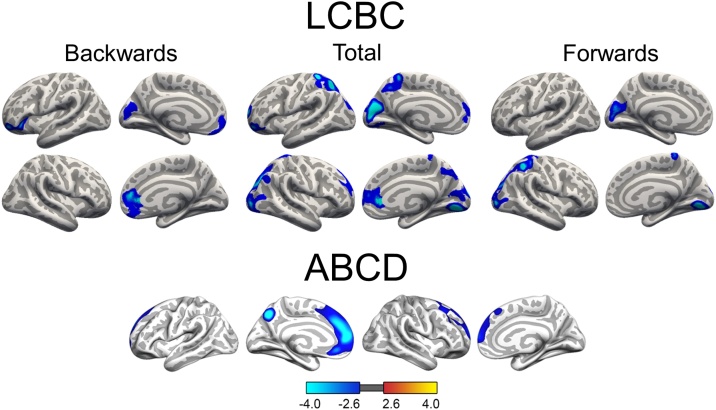
Relationship between working memory and cortical thickness during development. Significant, cluster-wise corrected, clusters from linear mixed models on vertex-wise thickness analyses. Blue-cyan indicates a negative relationship between thickness and WM scores. LCBC: The relationship between WM (indexed Digit Span Backwards, Digit Span Forwards and Digit Span Total) and cortical thickness, controlling for sex, age and scanner. The baseline age cut-oﬀ ; for the developmental group was ≤ 16.9 years. At follow up, the ages of these participants ranged from 4.4 to 26.7 years. ABCD: the relationship between List Sorting WM Test scores and cortical thickness (9.0–10.9 years), controlling for sex, age and ethnicity.

**Fig. 5:**
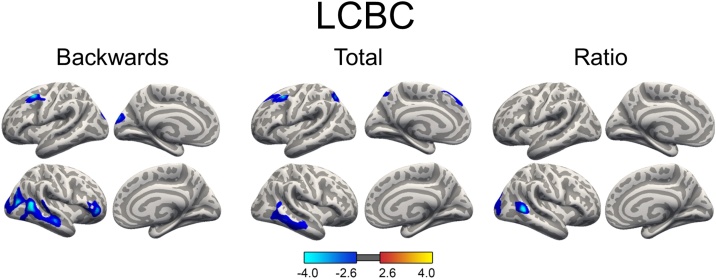
Relationship between Digit Span scores and cortical thickness during adulthood. Significant, cluster-wise corrected, clusters from linear mixed models on vertex-wise thickness analyses from the LCBC Adult sample, controlling for sex, age and scanner. The baseline age cut-oﬀ ; for the adult group was >16.9 years. At follow up, the ages of these participants ranged from 20 to 66.6 years. Blue-cyan indicates a negative relationship between thickness and WM scores. Total = Digit Span Total scores (forwards + backwards) and Ratio = ratio scores (backwards/forwards).

For the lifespan sample, linear mixed models were run on the cortical surface within the three age groups (development, adult and older adult) separately to test relationships between WM performance and cortical thickness. Age, sex and scanner were used as covariates of no interest. No WM-thickness effects reached significance for the older adult sample, while in the developmental and adult group, higher WM performance was related to thinner apparent cortex in widespread areas. For the developmental group, WM performance indexed by Digit Span Backwards scores was associated with thinner apparent cortex in prefrontal regions in both hemispheres and in left occipital lobe (see Fig. 4). Age-trajectories for these four regions showing a WM-apparent thickness relationship are illustrated in supplementary material, Fig. 2. Apparent thinner cortex in frontal regions in both hemispheres and in left occipital lobe was also found in association with Digit Span Total scores, in addition to apparent thinner cortex in bilateral parietal regions (see Fig. 4). The WM-thickness effects measured by the Digit Span Total scores and Digit Span Forwards scores were anatomically overlapping, with less extensive effects for the Forward condition (see Fig. 4). No association was found between apparent cortical thickness and Digit Span Ratio scores in the developmental group. GAMMs were also run to estimate an age-trajectory for mean cortical thickness for the LCBC Lifespan sample, covaried for sex and scanner. For the adult group, Digit Span Backwards, Total and Ratio scores were all associated with thinner cortex in middle temporal gyrus (Fig. 5). In addition, Digit Span Backwards and Digit Span Total were also associated with thinner cortex in frontal regions. The negative WM-thickness relationship found in the adult age group was surprising. Therefore, post hoc analyses were run to test the relationship between Digit Span Backwards and cortical thickness, dividing the adult age group into participants aged: 1) 20–39.9 years (n = 588 with 600 observations, and 2) 40–59.9 years (n = 153 with 216 observations). The results showed that the negative WM-thickness relationship was driven by the youngest adults aged 20–30.9 years, and no effects survived statistical correction for the age group 40–59.9 years (see supplementary material Fig. 3).

**Fig. 6:**
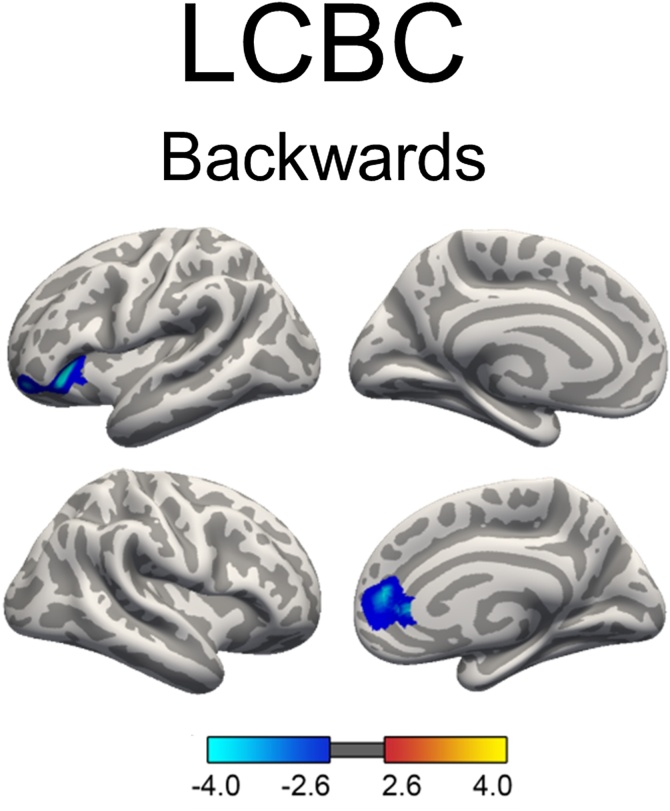
Relationship between working memory and cortical thickness during development, controlling for general cognitive ability. Results from LMEs showing relationship between Digit Span Backwards scores and cortical thickness, controlling for sex, age, scanner, and general cognitive ability scores as an additional covariate. The baseline age cut-oﬀ ; for the developmental group was ≤ 16.9 years. At follow up, the ages of these participants ranged from 4.4 to 26.7 years.

To control for effects of general cognitive function, we re-ran the analysis for Digit Span Backwards in the developmental sample, adding GCA scores as an additional covariate. The results showed a similar WM-thickness relationship only with less extensive effects, indicating that the majority of the effects could not be accounted for by general cognitive function (see Fig. 6). We also re-ran the analysis for Digit Span Backwards with mean thickness as an additional covariate to test for the specificity of the regional effects. No effects reached significance, suggesting that the observed effects to a substantial degree were due to a global effect of cortical thickness. Results from the supplementary analyses showed no effect of Digit Span Backward scores on volume for the three age groups, while a positive relationship was found between Digit Span Backward scores and surface area for the developmental and adult age group (see supplementary material, Fig. 4). Like for most of the cortical thickness relationships, the surface area relationships did not survive statistical correction when mean surface area was added as an additional covariate.

#### 3.2.2 ABCD sample

For the ABCD sample, GLMs were run to test the relationship between WM and cortical thickness, controlling for sex, age and ethnicity. Higher WM performance was related to thinner cortex in prefrontal and parietal regions (see Fig. 4). The anatomic overlap between WM-thickness effects from the ABCD and LCBC developmental samples, were mainly apparent in the medial orbitofrontal region, evidenced by a correlation of r = 0.19 (CI ± 0.005, p < .001). There were no significant relationships between WM performance and volume or surface area.

### 3.3 Co-heritability and socioeconomic status

To estimate how much of the relationship between WM and cortical thickness was due to common genetic influence, co-heritability was calculated for the three anatomical regions found to be associated with WM development in the ABCD sample (see Supplementary material Fig. 5). Results showing SNP-based heritability (h^2^) for each trait and co-heritability (co-h^2^) between WM and the thickness of the three regions are presented in Table 4. h^2^ for cortical thickness was modest, ranging from 0.117 to 0.212, and h^2^ for WM was 0.083. The results did not indicate a strong shared additive genetic component underlying the observed phenotypic WM-thickness correlation.

**Table 4:** Heritability and co-heritability for ABCD.

Variables	h2	SE	co-h^2^	p-value
WM	0.083	0.044		
Cluster 1	0.212	0.045	0.005	0.492
Cluster 2	0.117	0.043	−0.278	0.199
Cluster 3	0.165	0.044	−0.012	0.483

The table shows heritability (h^2^) for each trait and co-heritability (co-h^2^) between working memory (WM) and cortical thickness of the three anatomical regions. SE = standard error. P-values refer to the co-heritability.

In addition to estimating co-heritability for the observed phenotypic relationships, we also tested if the SES variables parental education and parental income affected the observed WM-thickness relationships in ABCD. Results showed that including the SES variables as additional covariates did not improve model fit compared to the regression model without SES variables (see Table 5). Thus, SES did not affect the relationship between cortical thickness and WM.

**Table 5:** Comparison of models with and without SES variables included for ABCD.

Clusters	res.df	rss	df	sumsq	F	p-value	AIC
Cluster 1	7982	144.383	–	–	–	–	−9375.027
Cluster 1	7980	144.270	2	0.113	3.132	0.044	−9377.279
Cluster 2	7982	271.255	–	–	–	–	−4336.618
Cluster 2	7980	271.175	2	0.081	1.186	0.305	−4334.994
Cluster 3	7982	169.208	–	–	–	–	−8107.336
Cluster 3	7980	169.128	2	0.081	1.907	0.149	−8107.154

Model fit of regression models with and without SES variables (parental education and parental income) as predictors. The first row for each cluster is a model without SES predictors, and the second is a formal model comparison of that model to a model with SES predictors. AIC = Akaike information criterion.

## 4 Discussion

We found that WM capacity develops throughout childhood and adolescence, declines at a low speed during adulthood, with accelerated decline in older adults. Interestingly, the trajectories for forwards and backwards, which presumably reflect short-term storage vs. active manipulation of information, respectively, showed very similar trajectories. WM development was related to apparent thinner cortex during childhood and adolescence, both in the LCBC and ABCD samples. The effects were spread across the cortical surface in frontal, parietal and occipital regions, and did not survive control for mean cortical thickness. This suggests that structural cortical maturation relevant for WM is not necessarily restricted to specific regions or networks. There was no strong shared additive genetic component underlying the observed WM-thickness relationship in development, and the effects were not mediated by SES. Implications of the results are discussed below.

### 4.1 Verbal working memory performance throughout the lifespan

In accordance with previous research, our results show that WM capacity increases during childhood and adolescence (Chiappe et al., 2000; Gathercole et al., 2004; Klingberg, 2006) (for a comprehensive review, see (Froudist-Walsh et al., 2018)), and decreases with age in adulthood (Gick et al., 1988; Hester et al., 2004; Kumar and Priyadarshi, 2013; Martial et al., 1994; Nyberg et al., 2012; Park et al., 2002; Wilde et al., 2004). WM performance was best described by three main phases of development and decline; rapid development until late teens, a longer period of relative stability in adulthood, followed by accelerated decline in higher age. Compared to other cognitive functions, such as episodic memory, we know less about the onset of decline in WM performance. Longitudinal studies of cognitive abilities that are highly related to WM performance have suggested a relatively late onset of average age-related decline, maybe after age 55 (Nyberg et al., 2012). The present results fit this view but suggest an even later age of onset of decline. The exact ages differentiating these general phases will likely vary with the type of test used to measure WM (Froudist-Walsh et al., 2018). Still, an interesting finding was that the age-trajectories for Digit Span Forwards and Digit Span Backwards were highly similar, and the correlation structure between the sub-measures was maintained across development and adult groups. The ratio (backwards/forwards), assumed to more purely measure the manipulation component of WM, controlling for the short-term storage component, changed during development only, with little vulnerability to aging.

### 4.2 Cortical changes are associated with verbal working memory

In both developmental samples, we observed a negative relationship between WM performance and apparent cortical thickness in extended regions. Thinner cortex has been found to be related to better cognitive performance in school-age years in a variety of cognitive domains in cross-sectional studies (Schnack et al., 2015; Squeglia et al., 2013; Tamnes et al., 2011; Østby et al., 2012), but see (Karama et al., 2011; Menary et al., 2013), including WM (Darki and Klingberg, 2015; Tamnes et al., 2010), and Digit Span performance (Kharitonova et al., 2013; Østby et al., 2011). The negative relationships with cognition reflect that apparent cortical thickness is reduced in this age range as a consequence of brain maturation. Few longitudinal studies on the relationship between WM and cortical structural change exist, with one study detecting significant cross-sectional effects only (Darki and Klingberg, 2015). In the other end of the lifespan, a longitudinal study of middle-aged and older adults did not find significant relationships between voxel-based morphometry measures of gray matter volume and n-back WM performance (Salami et al., 2018).The present study was very well powered to detect developmental relationships, and the mixed model approach takes advantage of both longitudinal and cross-sectional data, which may explain that we observed widespread negative WM-thickness relationships in development.

The WM-apparent thickness effects in development were relatively widespread, and did not survive inclusion of mean cortical thickness as covariate, suggesting that the effects are global rather than local. Even though there may be a mechanistic relationship between structural brain maturation and aging and changes in WM performance, it is not realistic to expect an anatomical one-to-one correspondence as structure-function relationships in general, especially in healthy groups, tend not to be strong (Van Petten, 2004). Successful completion of a WM task will depend on a range of different processes in the brain, where some will be more specific to WM and some more generally involved in demanding cognitive tasks, i.e. attention and control functions. Using functional imaging, contrasts can be designed to isolate such specific effects, but this is not feasible when testing relationships between behavioral and structural brain imaging data. In development and aging, WM performance will depend on all of these general and specific processes. Still, the observed WM-apparent thickness effects were present after general cognitive abilities were accounted for, which implies cognitive specificity for working memory. In the current study, two different WM tests were used, which also served as a validation of the test-specificity of the observed effects as the WM-thickness relationship showed the same directionality and somewhat anatomical overlap between the two samples. Still, not surprisingly, the results were not identical between the two samples, as ABCD had a much narrower age range, larger sample size, and only cross-sectional data available.

The negative WM-apparent thickness relationship in the adult age group was surprising. However, results from post hoc analyses showed that the relationship was driven by the youngest adults (< 40 years). The underlying mechanisms of apparent thinning are complex and believed to involve synaptic pruning as well as intracortical myelination (Huttenlocher, 1979, 1990; Huttenlocher and Dabholkar, 1997). By using a variety of different MRI modalities, additional information about cortical maturation can be obtained (Glasser and Van Essen, 2011), and increased myelin during development has been suggested to change the gray–white matter contrast in MR images resulting in apparent cortical “thinning” (Natu et al., 2019). Shafee et al. (2015) used the T1 / T2 ratio to estimate intracortical myelin content, and found that this metric increased from 18 to 35 years. Westlye et al. (2010) studied the variation in signal intensities that can be found in T1-weighted MR images and showed that while cortical thickness was negatively related to development, intracortical gray matter and subcortical white matter signal intensity increased until almost 30 years, before age-related reductions were evident. Combining the thickness measure with intensity measures might yield a more complete picture of cortical maturation.

While the present study had good power to detect WM-apparent thickness relationships in development and young adulthood, the power was smaller for the older age-range. For the oldest adults (> 60 years), uncorrected effects showed a positive WM-thickness relationship, as expected, but caution should be made when interpreting these results as they did not survive proper correction for multiple comparisons. Still, they confirm that the negative thickness-WM relationship is confined to the younger adults only. Also, Walhovd et al. (2020) have recently shown that grey matter volume is a better proxy for the brain foundations of general cognitive abilities compared to apparent cortical thickness in adulthood.

### 4.3 Co-heritability and socioeconomic status

We did not find evidence for a strong shared additive genetic component underlying the observed phenotypic correlation in ABCD. This could be due to none-additive genetic components and environmental factors playing a larger role. However, even with almost 8000 participants, the sample size could have been too small given the modest phenotypic correlation. Larger samples with both pedigrees and genotypes may help disentangle contributions from genetic and environmental components. Still, importantly, we estimated the SNP-based narrow-sense heritability, which is expected to yield lower estimates than twin-based heritability. Traditional twin studies estimate both genetic and non-genetic components in the models, while the current study only estimated the additive genetic component. Thus, the currently used SNP-heritability is a much more conservative estimate than the often-used twin-based heritability, which may explain the weak genetic contribution observed (Visscher et al., 2014).

As mentioned, the weak genetic effects could be due to strong environmental effects on the measures, thus, possible effects of SES were also tested in ABCD. Our results showed that the thickness-WM relationship was not mediated by parental education and parental income. This is in line with the lack of thickness-SES relationship reported in Judd et al. (2020). Thus, the thickness-WM relationship was not caused by a common influence of parental income or education on cortical thickness or WM performance, or any other genetic or non-genetic factors associated with this.

### 4.4 Limitations

Data from different scanners were used in both samples, which could potentially influence the results. However, validation analyses were conducted on the LCBC Lifespan data, suggesting that our approach is valid. Also, although longitudinal data was used this is still an observational study, and the association found does not imply casualization (Ghisletta et al., 2015).

## 5 Conclusion

WM performance has a protracted developmental course, but is relatively resistant to decline until relatively late in adulthood. This was especially evident for the active information manipulation component of WM. The development of WM performance was negatively related to apparent cortical thickness in two independent developmental samples, not restricted to known WM-networks. However, we did not obtain evidence for the genetic or the SES contributions to the relationship, which will await further studies.

## 6 Data availability statement

Ethical restrictions imposed by the Regional Committee for Medical and Health Research Ethics (REC South East Norway) as well as data storage requirements in accordance with Norwegians Laws of privacy protection does not allow for public availability of participant data. However, anonymized data will be made available upon request to individual researchers, pending ethical approval from REC. Interested researchers may contact project leader Prof. Kristine B. Walhovd (k.b.walhovd@psykologi.uio.no), who will seek permission to share this data.

## Declaration of Competing Interest

The authors report no declarations of interest.
