# Supplementary material for: Relationships between apparent cortical thickness and working memory across the lifespan - Effects of genetics and socioeconomic status
Source: Dev Cogn Neurosci. 2021 Aug 8;51:100997. doi: 10.1016/j.dcn.2021.100997 (PMC8371229; doi:10.1016/j.dcn.2021.100997)
Supplement: Supplementary file 1 [file mmc1.docx]

**Supplementary material**

| Ethnicity | Male | Female |
| --- | --- | --- |
| White | 2371 | 2079 |
| Hispanic | 1027 | 955 |
| Black | 680 | 640 |
| Other | 480 | 437 |
| Asian | 103 | 111 |
| Undisclosed | 6 | 4 |

*Table 1. Self-reported ethnicities for ABCD.*

Overview of the number of participants with different self-reported ethnicities. Undisclosed ethnicity means participants do not have ethnicity data provided.

*
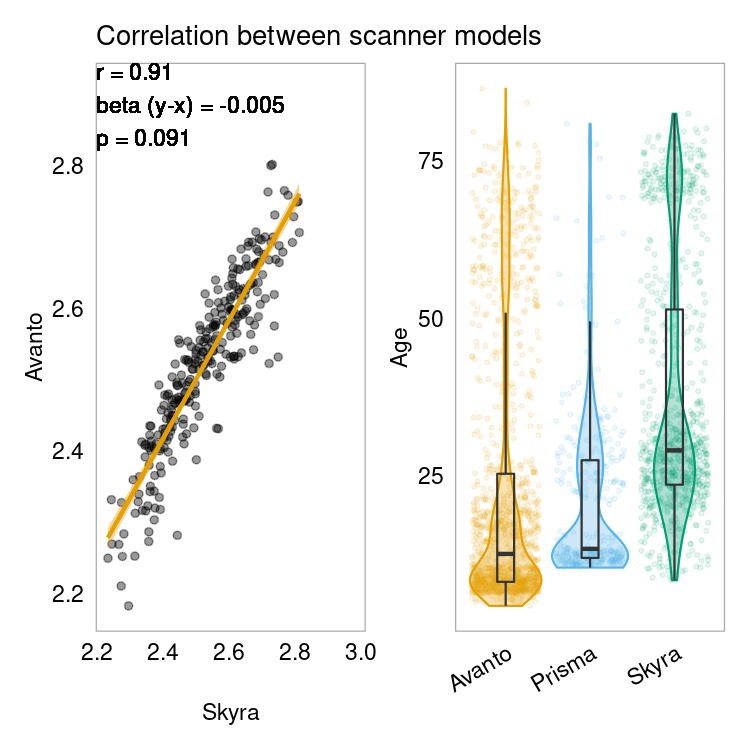
*

*Figure 1: Validating mean cortical thickness across different MRI scanners for LCBC.*

Left: The plot shows mean cortical thickness as a function of scanner. Simple correlations are tested by linear models predicting mean cortical thickness on the Avanto scanner using mean thickness from the Skyra scanner for the 307 participants that were scanned on both scanners on the same day. Right: Sample density is plotted as a function of scanner to view the age-range of samples from the different MRI scanners.


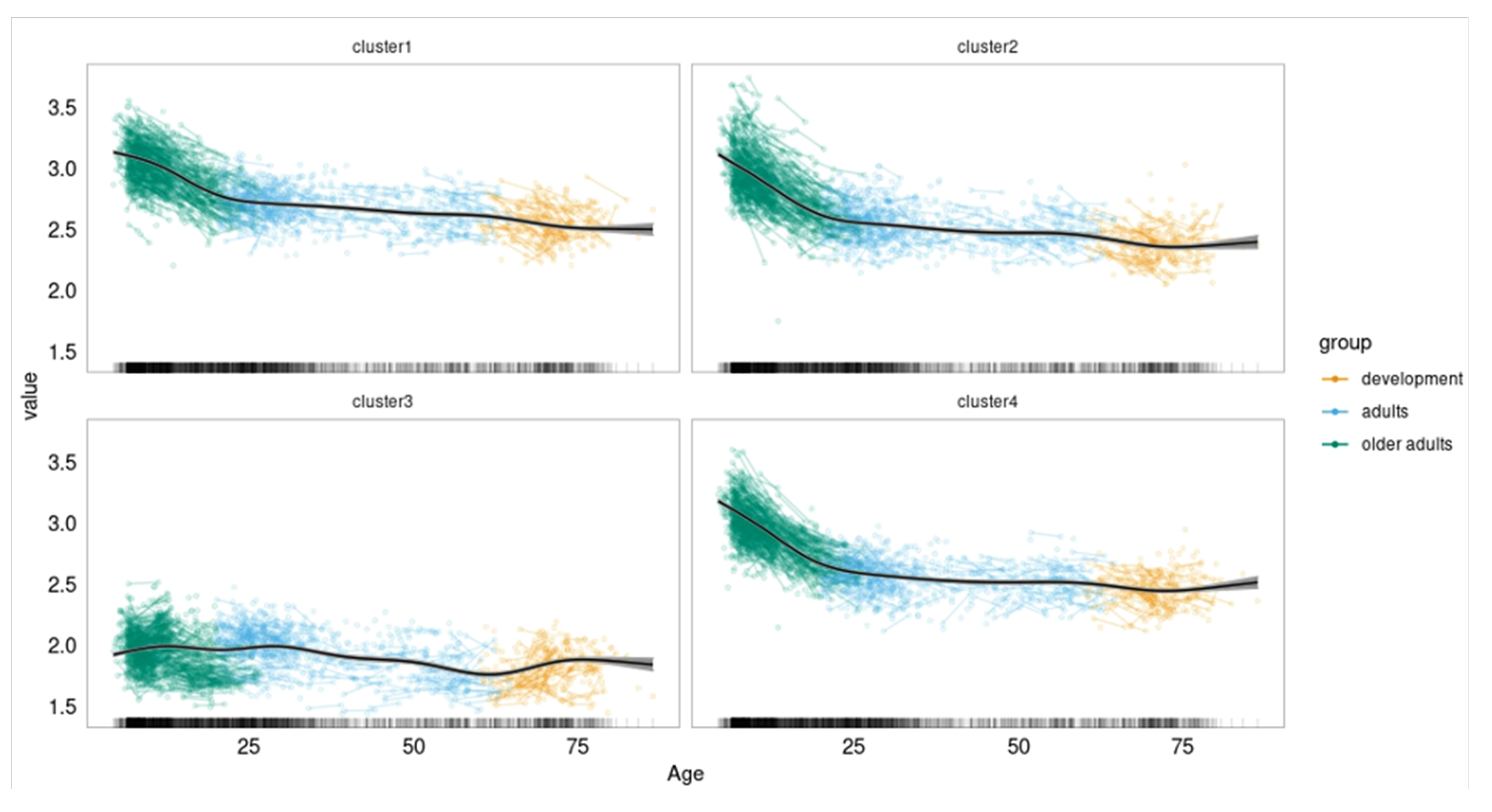
*Figure 2: Cortical thickness across the lifespan for regions showing WM-apparent thickness relationship during development*

LCBC Lifespan sample (4.4-86.4 years). The GAMM controlled for the effect of sex and scanner as covariates. The clusters refer to the four ROIs showing a WM-apparent thickness relationship in the developmental group. Working memory (WM) is here indexed by Digit Span Backwards scores. Cluster 1 = left rostral middle frontal, cluster 2 = left superior frontal, cluster 3 = left cuneus, cluster 4 = right rostral anterior cingulate cortex. The baseline age cut-oﬀ for the adult group was > 16.9 years of age, and > 59.9 years of age for the old adults.


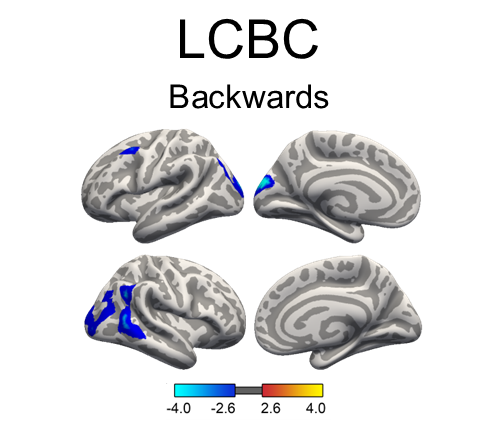


*Figure 3: Relationship between working memory and cortical thickness during adulthood (20 to 40 years).*

Results from post hoc analyses showing relationship between Digit Span Backwards scores and cortical thickness, controlling for sex, age and scanner, for the youngest adults from 20-30.9 years of age.


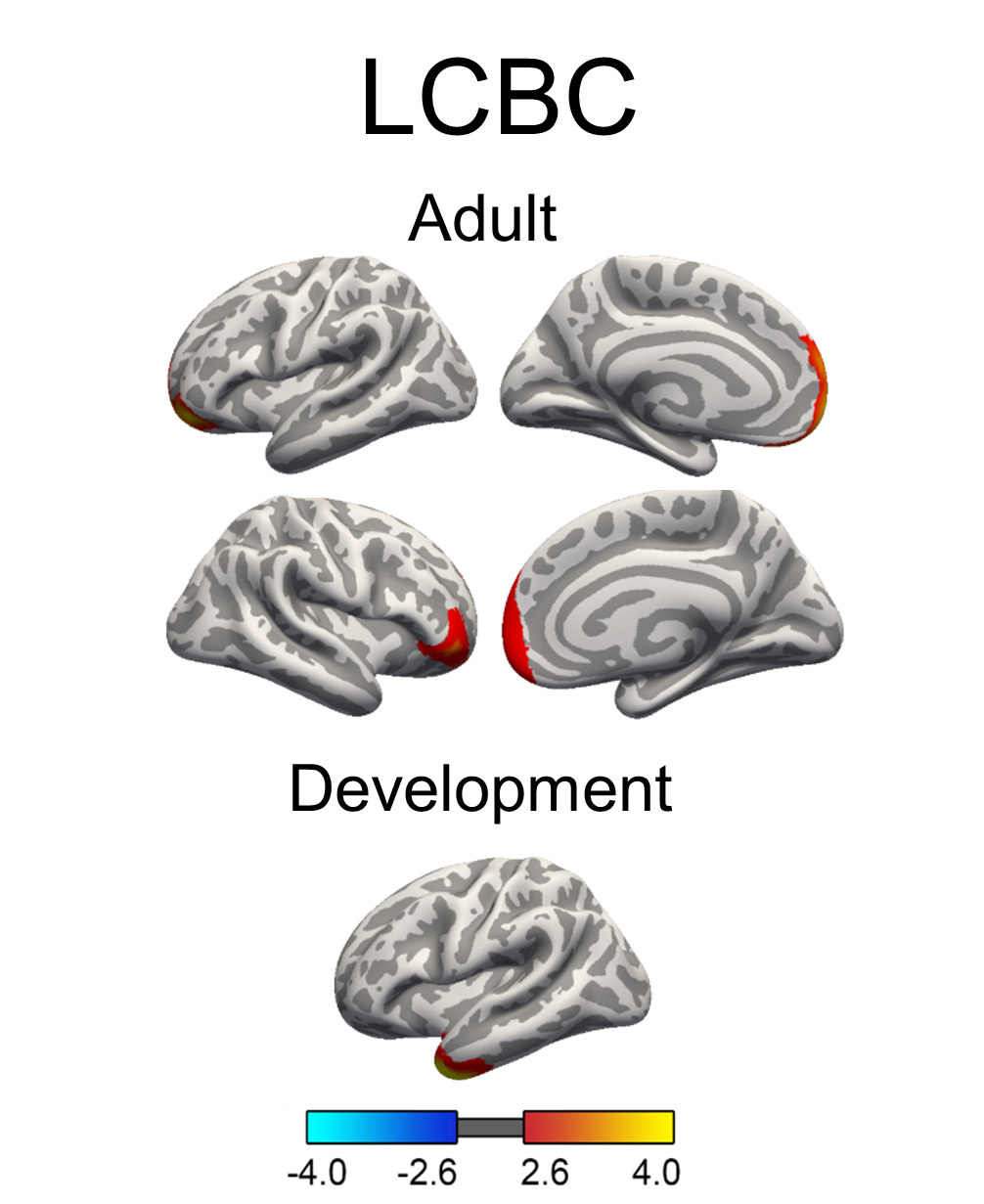


*Figure 4: Relationship between working memory and surface area during development and adulthood.*

Significant, cluster-wise corrected, clusters from linear mixed models on vertex-wise surface area analyses, controlling for sex, age and scanner. The red-yellow indicates a positive relationship between surface area and Digit Span Backwards scores. For the developmental group, the baseline age cut-oﬀ was ≤ 16.9 years. At follow up, the ages of these participants ranged from 4.4–26.7 years*.* The baseline age cut-oﬀ for the adult group was > 16.9 years. At follow up, the ages of these participants ranged from 20 – 66.6 years.


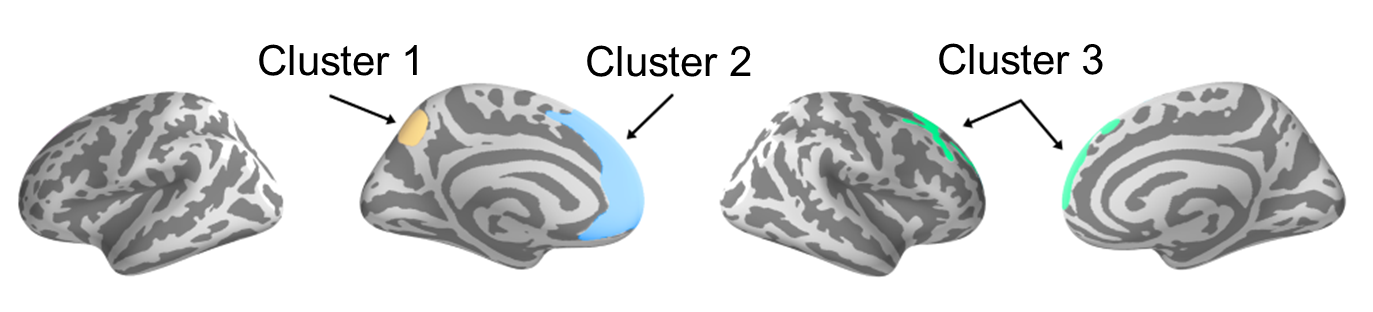


*Figure 5: ROIs used to estimate co-heritibality and SES.*Significant clusters from the vertex-wise linear mixed models for the WM-thickness effects for ABCD.
